# Effects of fertilisation on grass and forb gamic reproduction in semi-natural grasslands

**DOI:** 10.1038/s41598-021-98756-5

**Published:** 2021-09-27

**Authors:** Michele Scotton, Valentina Rossetti

**Affiliations:** Department of Agronomy, Food, Natural Resources, Animals and Environment, University of Padova, Viale dell’Università 16, 35020 Legnaro, PD Italy

**Keywords:** Plant ecology, Plant reproduction, Ecology, Plant sciences

## Abstract

Studying the effects of fertilisation on the seed production of grassland species can help understand the vegetation changes and biodiversity losses due to soil eutrophication. The seed production of fifteen grasses and seventeen forbs from a temperate hay meadow was studied under three fertilisation treatments: 0-0-0, 0-54-108 and 192-108-216 kg N, P_2_O_5_ and K_2_O respectively, per year. Fertile shoots collected at the seed maturation stage were analysed for all main traits of the gamic reproduction. On average, forbs produced more ovules and viable seeds per shoot (199 and 65, respectively) than grasses (112 and 35, respectively). Fertilisation increased the number of inflorescences per shoot in both grasses and forbs and had a limited but variable effect on germinability and viability in the two functional groups: viability increased in grasses but often decreased in forbs. This pattern resulted in 55% and 11% increases in viable seed production in grasses and forbs, respectively. At the higher level of fertilisation, shoot density was positively related to the number of viable seeds per shoot in grasses and to the seed size in forbs. These results highlight that the traits of the gamic reproduction can contribute to explain the relationship between soil nutrient richness and grassland species composition and richness.

## Introduction

Extensively managed semi-natural grasslands have high plant and animal richness and play a central role in the conservation of biodiversity; the rapid decrease in these habitats in the last few decades is of great concern for European environmental policy^1^.

An issue critical for the conservation and restoration of semi-natural grasslands is the seed production of the species that compose them. In-depth study of the gamic reproduction of grassland species could reveal why grassland species react to different cultivation practices and clarify the degree to which grassland vegetation evolves following changes in management^2^. The effects of anthropogenic increases in terrestrial soil nutrient concentrations^3^ must be investigated to clarify the possible effects of soil eutrophication on the biodiversity and functioning of grasslands. The persistence of forbs is at particular risk owing to soil eutrophication as forbs reproduce primarily by seed, whereas most grasses can reproduce through both agamic and gamic reproduction, propagate through tillering^4^, and greatly benefit from high soil nutrient levels^5^. The patterns of grassland species seed production are also crucial knowledge for planning efficient seed harvests aimed at restoring species-rich grasslands^6^.

At the fertile shoot level, the output of gamic reproduction is the amount of viable seeds produced during a plant’s life. This quantity is influenced by several reproductive traits: number of inflorescences per shoot, flowers per inflorescence, and ovules per flower, which determine the number of ovules per shoot (i.e., the seed yield potential); the proportions of ovules transformed to filled seed (seed set), and filled seeds which are viable (fulfilment of the yield potential)^7^.

The above-mentioned traits are primarily genetically determined but can also be influenced by environmental factors^8^. The influence of soil fertility has received particular attention because of its importance for seed propagation and, more recently, its increase in terrestrial soils as a consequence of human activities^3^. Past studies have highlighted the degree to which soil fertility can affect the individual traits that determine seed yield. In grasses, for example, high nitrogen (N) supply can enlarge the inflorescence by increasing the number of flowers per spikelet or the number of spikelets per shoot^8^. High phosphorous (P) and potassium (K) availability was found to indirectly enhance flower formation through increasing the concentration of cytokinins in the plant^9^. Stephenson^10^ reported that seed set was generally increased by higher availability of the macronutrients necessary for fertilised ovules to develop into seeds. However, Wiens^11^ found that seed set was more dependent on genetic factors. Fertilization was also reported to influence the pollinator behaviour in outcrossing species^12^. In the grassland species *Gymnadenia conopsea*, NPK addition increased the time spent by pollinators at each plant due to a higher nectar amino acid content^13^. In *Allium cepa*, NP fertilisation reduced the number of pollinator visits probably due to a lower nectar attractiveness^14^. In both cases, increased selfing was observed with a consequent reduction of seeds with embryo^13^ and seed set^14^. Fenner^15^ reported that seed size and mineral concentration are not typically affected by soil fertility, so that even in infertile soils, seedling recruitment is not negatively affected.

The gamic reproduction of temperate grassland species has primarily been researched within the field of seed propagation, where fertilisation is the primary agronomic practice applied to influence seed yield^16^. Moderate fertilisation rates are typical to avoid an excess of vegetative growth that competes energetically with reproductive development^17^, and one, two, or all three primary macronutrients may be added, dependent upon the species being propagated and the base soil fertility. In semi-natural grasslands, fertilisation is also the management strategy with the strongest influence on grassland traits^18^. Fertilisation levels have widely varying effects on gamic reproduction strategies and on seed yields, which can vary greatly by species, quantity of fertilizer, and time of distribution^6^. However, these effects can diverge significantly from those found in the propagation of forage seed, for the following reasons:

1. grasslands are composed of many species with contrasting phenological strategies and different reactions to the same fertilisation;
2. different aims of grassland cultivation (forage vs. seed yield) entail different management practices (e.g., earlier harvesting to obtain higher-quality forage);
3. in grasslands, fertilisation is typically organic and all macronutrients are applied together, often at a very high rate.

Despite the importance of gamic reproduction in grasslands, no comprehensive studies have been conducted at the community level to address the effect of fertilisation on the primary plant species and species functional groups of these ecosystems in temperate climates, so that it is difficult to make any research hypotheses except for the general assumption that the soil nutrient-enrichment could more favourably enhance the gamic production in grasses than in forbs. Therefore, this study focused on the gamic reproduction of an important type of temperate grassland with the intent to:

- analyse the reproductive traits of the main grassland species and the two functional groups (grasses and forbs), and quantify their response to fertilisation;
- identify biological and ecological traits explaining the response to fertilisation of the species gamic reproduction behaviours; and
- identify correlations among reproductive traits, defining different reproductive strategies and quantify their responses to fertilisation.

## Materials and methods

### Site, meadow, and fertilisation

The grassland utilized in this study was located in Sedico (BL: 420 m a.s.l., eastern Italian Pre-Alps), where the annual mean temperature is 10.6 °C and the annual rainfall is approximately 1366 mm (389, 326, 401, and 250 mm in spring, summer, autumn, and winter, respectively). The site was level and had an alluvial calcareous substratum. The soil was sandy-loam textured with 12.2% gravel content, 14.6% total carbonate content, and a pH of 7.5. Since 1977, a section of the meadow has been used for a fertilisation trial organised as three completely randomized blocks with 24 m^2^ plots and twenty-seven treatments obtained by combining three levels of yearly N, P, and K applications per ha: 0, 96, and 192 kg N as ammonium nitrate; 0, 54, and 108 kg P_2_O_5_ as triple superphosphate; and 0, 108, and 216 kg K_2_O as K sulphate. Since 2010, the grassland has been cut twice per year and surveyed for seed production in three treatments: no fertilisation (000), fertilization with no N and intermediate levels of P and K (011), and fertilisation with the highest nutrient rates (222).

The vegetation of the three treatments (Annex 1) corresponded to the following meadow types: type 000, vegetation intermediate between a poor-soil form of the *Arrhenatherum elatius* meadow (Ar0) and a *Bromus erectus* meadow (Br), with high species richness and low legume abundance; type 011, an Ar0 meadow with high species richness and legume abundance; and type 222, a grass-rich form of the *Arrhenatherum elatius* meadow with low species richness and legume abundance.

### Plant sampling and laboratory analysis

During the two growth periods within each of the years from 2012 to 2017, fertile shoots were sampled from the three fertilization treatments. 15–30 shoots (5–10 in each plot) from each flowering species were collected at the optimal seed maturation stage (most fruits/inflorescences still intact, i.e., no seed shedding). At the sub-plot level, all fertile shoots were collected at the time of meadow mowing on one 1-m^2^ sub-plot per plot. Collected shoots were put separately per species into porous paper bags, dried, and preserved in a refrigerator until laboratory analysis.

During the autumn and winter after collection, the 15–30 shoots of each species were analysed for the number of compound and/or simple inflorescences and the flowers per simple inflorescence or shoot. For species with flowers or inflorescences too numerous to be rapidly counted, an inflorescence length or diameter which could be related to the number of flowers was also measured (e.g., the panicle length in grasses). In sample flowers, intact fruits, or simple inflorescences, the number of ovules per flower and the number of ovules transformed to seed were observed under a binocular microscope. Mature seeds were weighed and tested for germinability and viability according to ISTA^19^. Germination trials were performed with three seed samples per species, which were placed on filter paper in petri-dishes and moved to a germinator for 4 weeks (8 h light/25 °C and 16 h darkness/15 °C) with weekly observation and extraction of germinated seeds. At the end of the germination test, seeds that had not germinated were checked for viability with the tetrazolium test. Total viability was calculated as the sum of germinability and viability of non-germinated seeds.

All shoots collected on the sub-plots were counted and measured for the number of inflorescences and flowers. When inflorescences and flowers were too numerous to be counted rapidly (e.g., in all grasses), only the same length/diameter measured on the 15–30 shoot samples was recorded.

A more detailed description of the laboratory analyses is available in Scotton^20^.

### Data analysis

The value of each reproductive trait was calculated for each year and growth period at the plot level for each species. The values of the traits describing the size of the reproductive system were obtained from the shoots collected on the sub-plots. However, for species with too many flowers per shoot, a relation was calculated between the flowers per shoot and the length/diameter of the inflorescences measured on the 15–30 shoot samples. This relationship was then used to calculate the flower number for each shoot. The number of ovules per flower, the portion of ovules transformed to seed (ovule site utilisation, i.e. the filled seed/ovule ratio), the 1000-seed weight, germinability, and viability were calculated from the results of lab analyses of the 15–30 shoot samples.

Because all the species collected were not always present in the six study years, only the thirty-two species (fifteen grasses and seventeen forbs: Table 1) found in at least three of the study years were considered in this paper to obtain enough reliable results. For all grasses, data were available only for the first growth period. For four forbs (see Annex 3) data were available for the first and second growth periods: in these cases, the average values of the two periods were used in the analyses.

**Table 1:** Species studied for reproductive traits in a grassland fertilisation trial in the Italian eastern Alps.

Grasses	Code	Fertilisation treatment (NPK level)			FORBS	Code	Fertilisation treatment (NPK level)		
		0 0 0	0 1 1	2 2 2			0 0 0	0 1 1	2 2 2
Anthoxanthum odoratum	AnOd	x	x	x	Achillea roseo-alba	AcRo	x	x	x
Cynosurus cristatus	CyCr	x	x	x	Clinopodium vulgare	ClVu	x	x	x
Dactylis glomerata	DaGl	x	x	x	Trifolium pratense	TrPr	x	x	x
Festuca pratensis	FePr	x	x	x	Centaurea nigrescens	CeNi	x	x	x
Holcus lanatus	HoLa	x	x	x	Rhinanthus freynii	RhFr	x	x	x
Trisetum flavescens	TrFl	x	x	x	Salvia pratensis	SaPr	x	x	x
Briza media	BrMe	x	.	.	Silene vulgaris	SiVu	x	x	x
Brachypodium rupestre	BrPi	x	x	.	Cerastium fontanum	CeFo	x	x	x
Avenula pubescens	AvPu	x	x	.	Medicago lupulina	MeLu	x	x	x
Festuca rupicola	FeRu	x	x	.	Ranunculus acris	RaAc	x	x	x
Arrhenatherum elatius	ArEl	.	x	x	Plantago media	PlMe	x	x	.
Bromus hordeaceus	BrHo	.	x	x	Primula veris	PrVe	x	x	.
Carex contigua	CaCo	.	x	x	Stachys officinalis	StOf	x	x	.
Lolium perenne	LoPe	.	x	x	Knautia drymeia	KnDr	x	x	.
Poa trivialis	PoTr	.	x	x	Leontodon hispidus	LeHi	x	x	.
–	–	–	–	–	Leucanthemum vulgare	LeVu	x	x	.
–	–	–	–	–	Rumex acetosa	RuAc	.	x	x

The graminoid species *Carex contigua* is included in the grass functional group.

The statistical analyses were performed at the levels of individual species and the two grassland functional groups (grasses and forbs). Nine main reproductive traits describing the whole process of gamic reproduction were considered: number of simple inflorescences per shoot, flowers per simple inflorescence, ovules per flower, ovules and viable seeds per shoot, OSU (ovule site utilization), percent viability, germinability, and seed weight. Percent dormancy (the difference between percent viability and germinability) and the shoot density recorded in the subplots were also considered in some analyses.

Only sixteen species were present in all of the fertilisation treatments, presenting a challenge in the tests that included all of the species together because a balanced among-treatments comparison was only possible by discarding the data from species not present in all of the treatments. To overcome this issue, we assumed that due to symbiotic N-fixation, the high presence of legumes in the 011 treatment (fertilization with P and K) was equivalent to a yearly N fertilisation of about 3.5 kg/ha per percent point of legume abundance in the species composition^21,22^. Therefore, treatment 011 (30% more legume abundance than in treatment 222: Annex 1) was regarded as an intermediate N addition of 105 kg per ha per year (from 3.5 kg N × 30% legume abundance). The values of the reproductive traits were then calculated for two fertilisation levels, low (LowFert) and high (HighFert). For species present in 000 and 011, LowFert was 000 and HighFert was 011. For species present in 011 and 222, LowFert was 011 and HighFert was 222. For species present in three fertilisation treatments, LowFert was 000 and HighFert was the average between 011 and 222. Statistical analysis considering only the species present in all fertilisation treatments yielded a similar pattern of fertilisation effects to those found in analysis of the two separate fertilisation levels. The analysis of the two fertilisation levels was therefore utilized because it was representative of a larger number of species.

Statistical analyses (see summary in Annex 2) were conducted with three main aims: 1. studying the fertilisation effect on the reproductive behaviour of individual species and the two species groups of grasses and forbs; 2. finding species biological and ecological traits explaining their response to fertilisation; and 3. identifying multispecies correlations among reproductive traits and the possible effects of fertilisation on their patterns.

For the first aim, the fertilisation effect was tested for the reproductive traits of each individual species through application of a mixed linear model under a repeated measure approach. In the model, fertilisation treatment, year, and block were input as class factors, and a plot identifier was used as a random factor. In case of significant fertilisation effects, the among-treatment differences were tested using the Tukey multiple comparison adjustment. Prior to performing the mixed model, data were checked for homoscedasticity and normality and, if necessary, log-transformed.

From the individual species mixed models, a table was calculated containing the frequency of cases with fertilisation effects (three levels: no, positive, or negative) for each reproductive trait and species group. To check if grasses and forbs differed for the obtained frequencies, for each trait a chi-square test on the frequency table “fertilisation effect x species group” was performed.

In a following set of analyses, the effect of the grassland functional group (grasses or forbs) on the multi-year means of each reproductive trait was tested with general linear models (GLM). Prior to the analysis, the data were sometimes log-transformed to mitigate homoscedasticity and normality problems. In these analyses, species were considered as replicates within the species group (therefore not included as a class factor) and the fertilisation level was input as a class factor. The effect of the fertilisation level on each reproductive trait was tested separately for the two species groups. In this case, the GLM included both fertilisation level and species as class factors.

For the second aim, possible biological and ecological traits explaining the species response to fertilisation were investigated by relating the percent variation due to fertilisation in two important size traits (ovule and viable seed number per shoot: variables Y) to the following explanatory (X) variables: average values of the nine reproductive traits, the seven Ellenberg bioindicator values^23^, and the percent variation of shoot density. The relationships were fitted according to a linear regression approach for grasses and forbs together or separately and checked for the parametric assumptions of residual normality and homoscedasticity. For the percent variation due to fertilisation of individual species of OSU, seed germinability, viability and weight, one-way analyses of variance were performed where three traits of the species reproductive biology (type of reproduction, breeding system and pollen vector^4^: Annex 1) were used as categorical factors. A GLM approach was also used in this case.

For the third aim, multispecies correlations were analysed by in-pairs relating the reproductive trait values of individual species averaged across fertilisation treatments and years. Fertile shoot density recorded in the subplots. was used as a supplementary characteristic. Nonlinear relationships were made linear with a log-transformation. Because the purpose of the analysis was not to predict one trait from the other but to efficiently summarise the relationships between traits, the standardised major axis (SMA) approach was used instead of the linear regression method^24^. The analyses were performed for grasses and forbs both together and separately. In order to verify if fertilisation could affect the characteristics of the evaluated relationships, a second set of SMA analyses were performed by separating the two fertilisation levels and the lines obtained were tested for common slope and elevation according to Warton et al.^24^.

The year effect will be reported in a forthcoming paper and is therefore not discussed here, despite its inclusion in the statistical analyses.

We used SAS/STAT software 12.3^25^ with procedures MIXED, GLM, REG, and UNIVARIATE, and R 3.0.0^26^ with package SMATR.

### Additional statements

The experimental research and the collection of plant material was done according to relevant institutional, national, and international guidelines and legislation. No grassland species considered in the research is included in the list of endangered species according to the IUCN, European Union, Italian national and regional classifications. The collection of plant specimen was done with permission of the grassland owner during the hay-making operations for forage production which do not need a special permission from the concerned local authorities. The plant species were identified by the first author, Michele Scotton. A voucher specimen of each plant species considered in the research was stored in the laboratory of the authors’ Department (DAFNAE) and the authors have provided an ID number for each voucher specimen.

## Results

### Frequency and amount of the fertilisation effect in grass and forb functional groups

**Figure 1:**
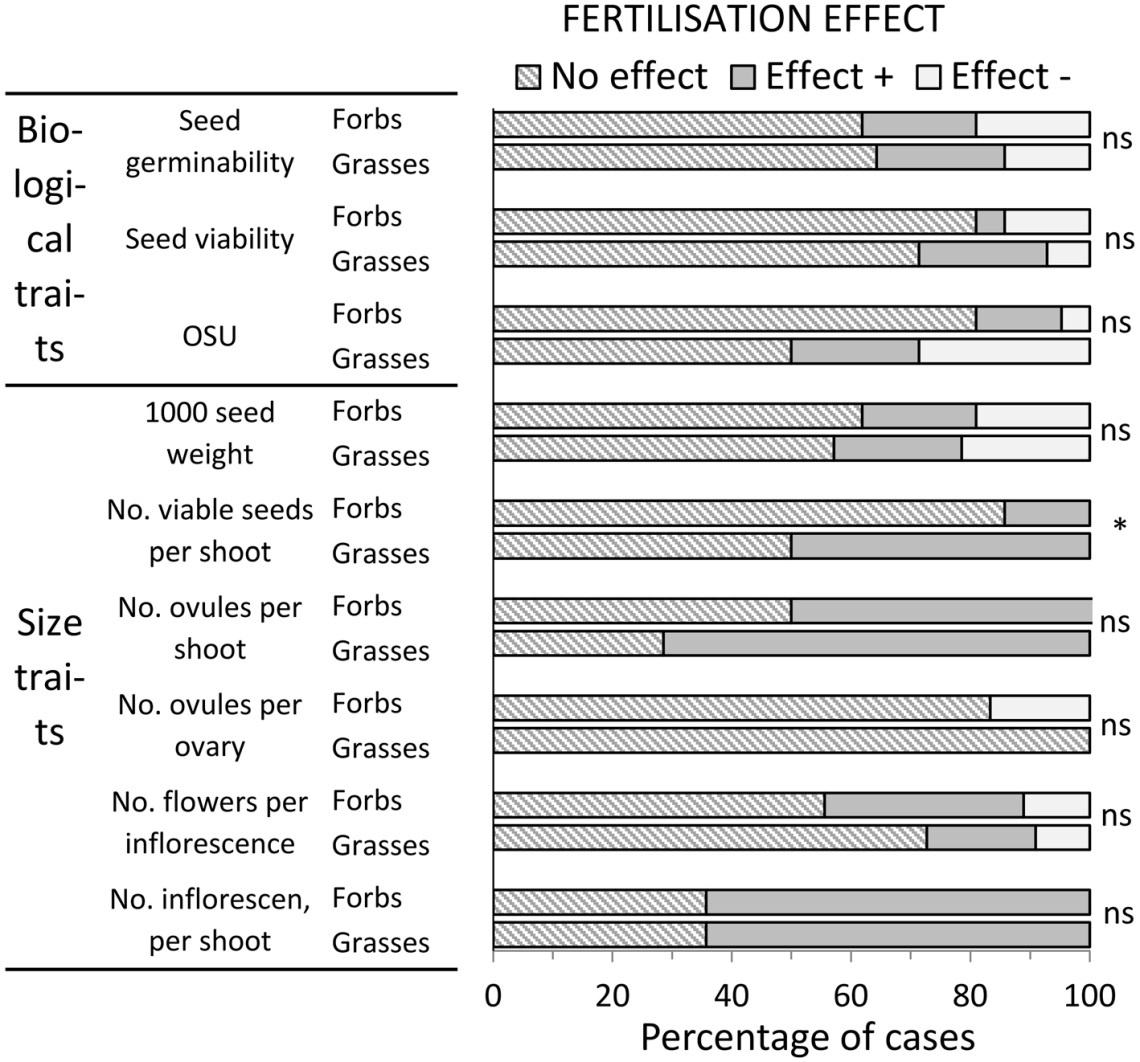
Frequency of different effects of fertilisation on the reproductive traits of fifteen grasses and seventeen forbs in a grassland fertilisation trial in the Italian eastern Alps. No effect, Effect + , and effect—denote statistical not significant, and positive or negative effects, respectively. ns and *indicate no significance or significance at p ≤ 0.05 of the chi-square test for the association between species group (grasses and forbs) and type of fertilisation effect.

The frequency of significant fertilisation effects was highly variable depending on the reproductive trait (Fig. 1 calculated from Annex 3). Fertilization effects were more frequently significant for the size traits of the reproductive system than for biological traits (OSU, viability, and germinability), and were generally positive. For biological traits, the effect was often both positive and negative. The seed quality traits (1000-seed weight and germinability) were frequently affected by fertilisation, both positively and negatively.

Comparing grasses and forbs, individual size and biological traits determining viable seed production per shoot did not display significantly different frequencies of responses to fertilisation, but the resulting viable seed production per shoot increased more frequently in grasses due to more frequent increases of ovules per shoot and no decrease in viability, in contrast to frequent decreases in viability in forbs.

**Figure 2:**
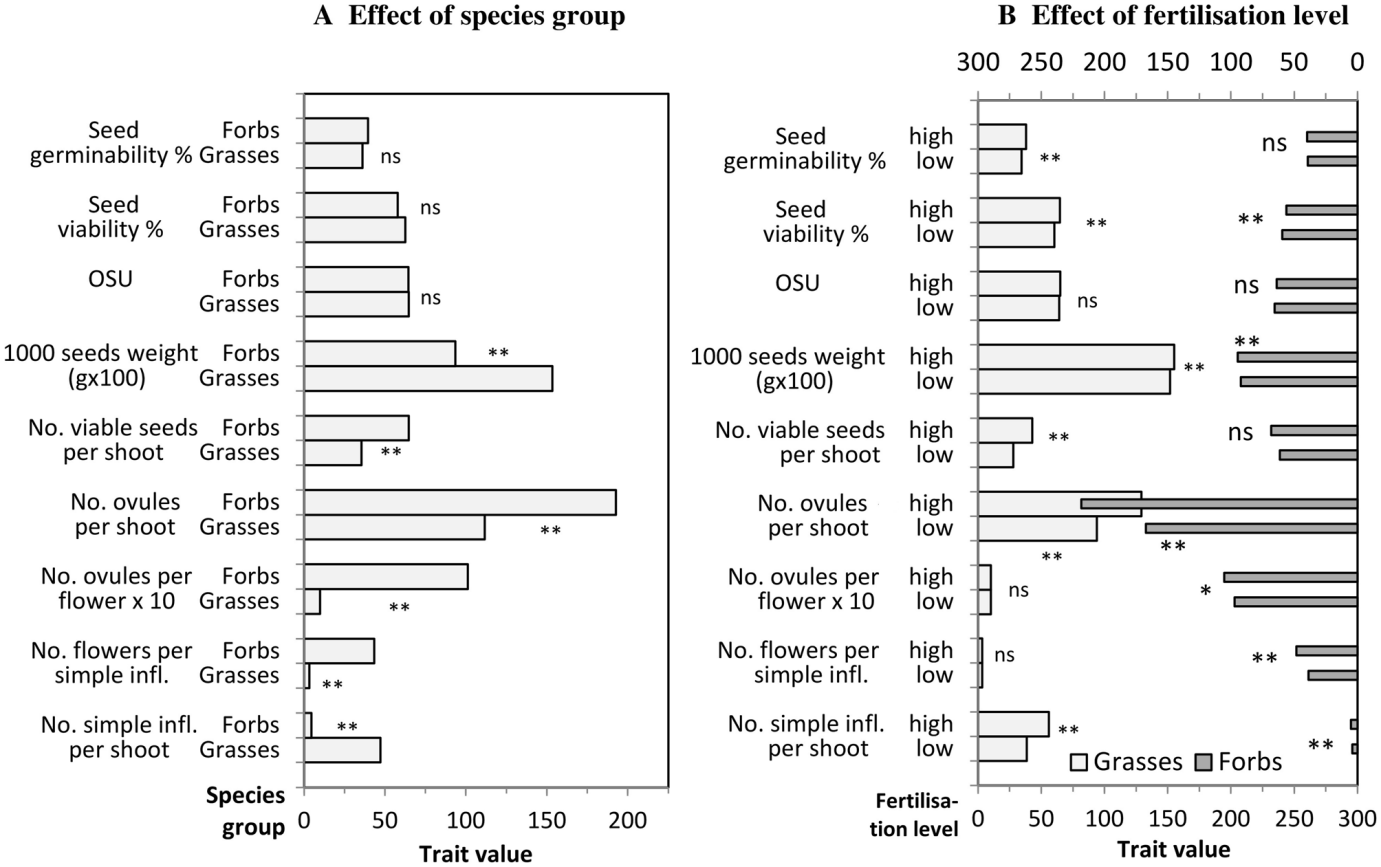
Mean values of the reproductive traits of fifteen grasses and seventeen forbs in a semi-natural grassland fertilisation trial. Graph A shows the comparison Grasse versus Forbs, graph B the comparison low vs. high fertilisation level within each functional group. ns, *, and **show the results of the Tukey’s test and mean not significant, significant at p ≤ 0.05, and significant at p ≤ 0.01, respectively. Individual species values are shown in Annex 3.

Grasses displayed a greater number of simple inflorescences per shoot than forbs (47 vs. 4: Fig. 2A), but fewer flowers per inflorescence (3.4 vs. 43) and ovules per flower (1 vs. 10). Overall, the number of ovules per shoot was significantly higher in forbs (193 vs. 112). OSU and viability did not differ between the two functional groups. Forbs produced on average more viable seeds per shoot than grasses (65 vs. 35). Percent dormancy (difference between percent viability and germinability) was significantly higher (26 vs. 18%) for grasses than for forbs (data not shown).

In grasses, fertilisation significantly increased the size of the reproductive system (Fig. 2B). The increase was exclusively due to a higher number of spikelets per shoot, which elicited a 37% increase in the number of ovules per shoot. Fertilisation also increased viability, but did not increase OSU. The resulting increase of the viable seed production per shoot was a positive gain of 54% (43 vs. 28 seeds). Fertilisation had a weak positive effect on the seed quality traits, increasing the seed size by 2% and the germinability by 10%. Dormancy was not affected by fertilisation (data not shown).

In forbs, fertilisation increased the number of ovules per shoot by 30% (Fig. 2B). However, viability in fertilized plants decreased significantly (5%), resulting in an overall increase in viable seeds per shoot of only 11% (68 vs. 61 seeds), a weaker effect than in grasses. Seed quality traits were also increased by fertilisation in forbs, but more weakly than in grasses. Due to the simultaneous decrease of viability and increase of germinability, dormancy was significantly reduced by 4% (data not shown).

### Relationships between fertilisation effects and species biological and ecological traits

**Figure 3:**
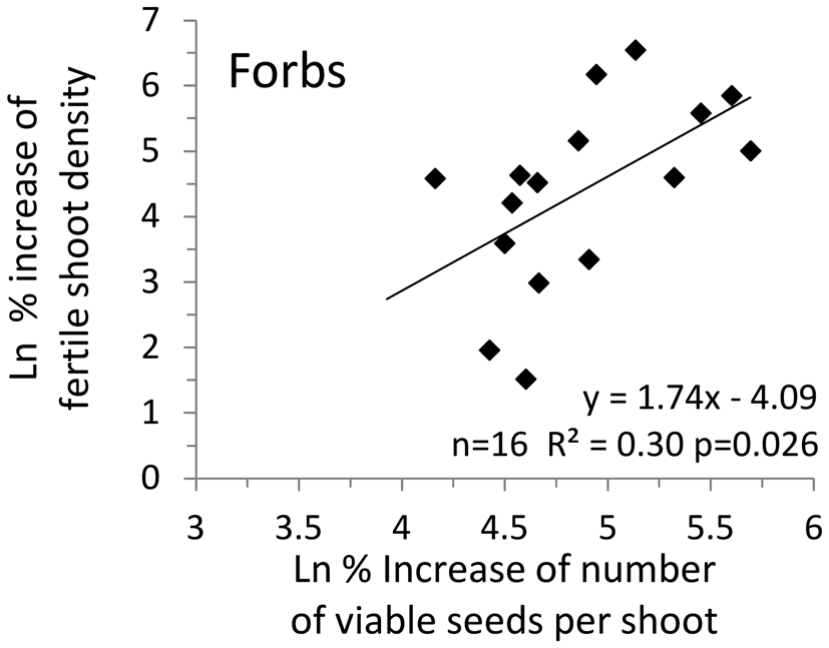
Relation between variation of number of viable seeds pr shoot at higher compared to lower fertilisation level and variation of fertile shoot density in seventeen grassland forbs. Data from a fertilisation trial in a semi-natural grassland of the Italian eastern Alps.

No relation was found between the percent variation of the number of ovules or viable seeds per shoot in HighFert as compared to LowFert (Y) and average values of reproductive traits or Ellenberg’s bioindicators (X). In forbs, a positive relationship was found between the percent increase of the fertile shoot density (Y) and of the number of viable seeds per shoot (X) in HighFert compared to LowFert (Fig. 3).

**Figure 4:**
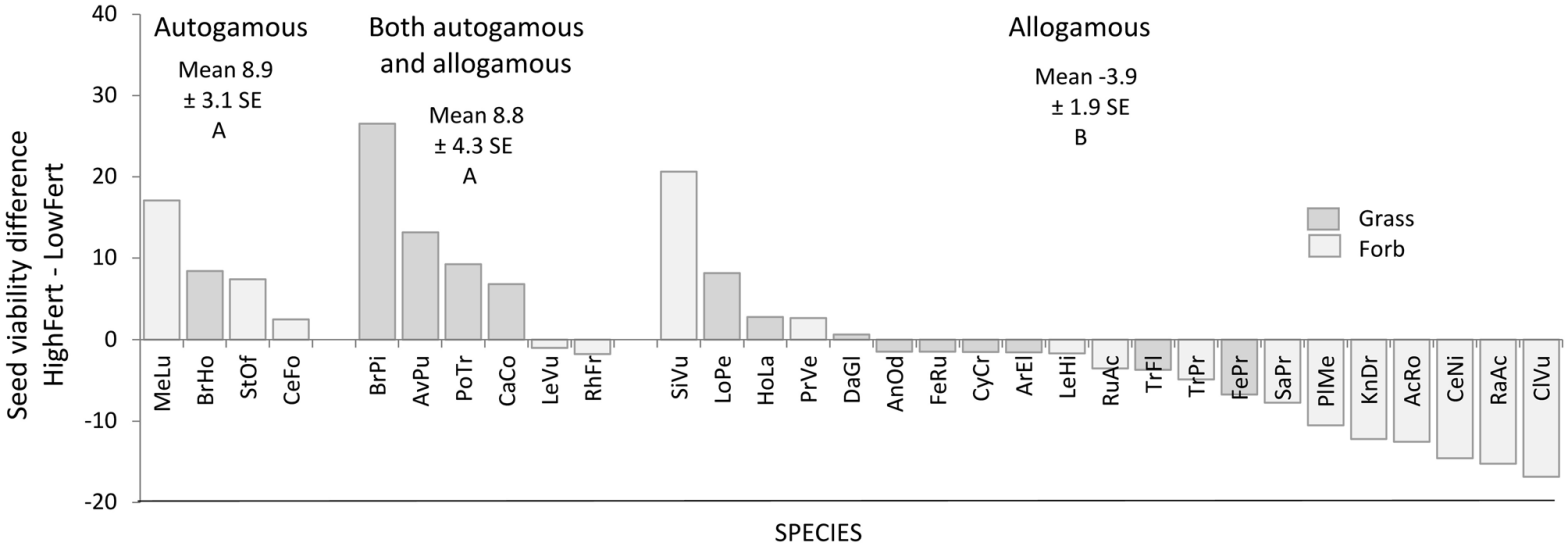
Relationships between variation of viability at higher compared to lower fertilisation level and breeding system of thirty-one grassland species. Data from a fertilisation trial in a semi-natural grassland of the Italian eastern Alps. Species codes in Table 1. Means with different capital letter differ at p < 0.01 according to the Tukey’s test.

Breeding system and species group significantly influenced the response of seed viability to fertilisation. Viability in HighFert was 9% higher than in LowFert for facultative or obligate autogamous species but 4% lower for obligate or primarily outcrossing species (p = 0.0032) (Fig. 4). Viability was also 4.2% higher for grasses and 3.1% lower for forbs (p = 0.05).

### Multispecies correlations among reproductive traits

**Figure 5:**
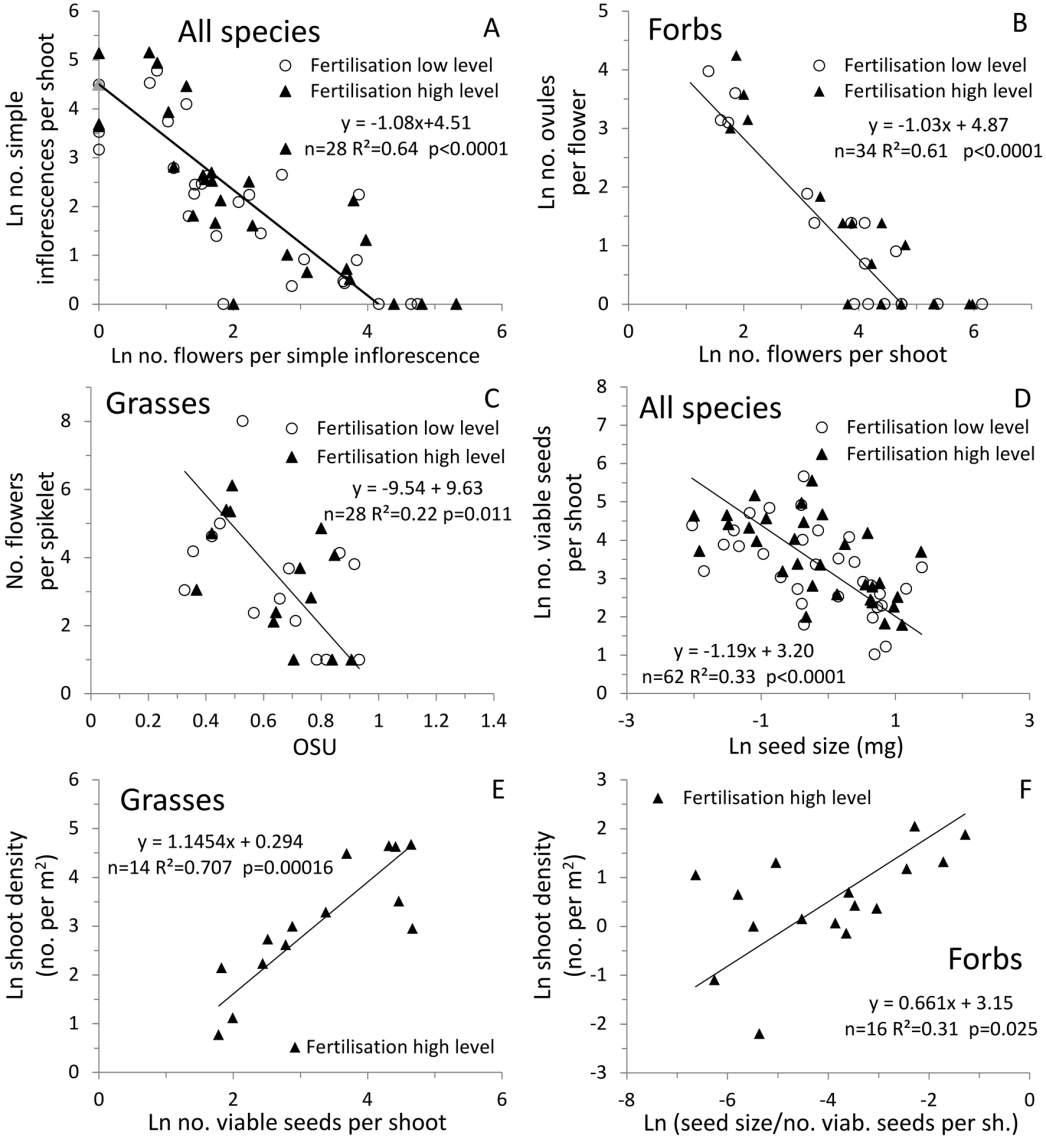
Inter-species relationships between reproductive traits of thirty-two grassland species based on data from a fertilisation trial in a semi-natural grassland of the Italian eastern Alps. Relationships shown are between traits at the fertile shoot level in graphs (**A**)–(**D**) and between a trait at the fertile shoot level and a trait at the population level (fertile shoot density) in graphs (**E**)–(**F**). Lines obtained with the standardized major axis method. In (**F**), the outlier value of *Cerastium fontanum* was excluded from the calculation of the line.

The following negative relationships were noted linking reproductive traits to each other: the number of simple inflorescences was related to the number of flowers per simple inflorescence in both grasses and forbs (Fig. 5A); the number of ovules per flower was related to the number of flowers per shoot in forbs (Fig. 5B); the number of flowers per spikelet was related to OSU in grasses (Fig. 5C), and; the number of viable seeds per shoot was related to seed size in both grasses and forbs (Fig. 5D).

Lines of best fit were power functions with negative exponents in 5A, 5B, and 5D, and linear in 5C. Relationships did never differ between the two fertilisation levels, therefore, only one common line was calculated. When fitting was performed separately, the line obtained from the HighFert level shifted more in the direction of the axis representing the trait most influenced by fertilisation. This effect was particularly evident for the seed size to seed number relationship (Fig. 5D) where the HighFert line was shifted upwards in the direction of the vertical axis representing the viable seed number which was affected by fertilisation much more than the seed size (horizontal axis).

In grasses, shoot density was positively related to the number of viable seeds per shoot (Fig. 5E) and, due to the inverse relationship between viable seed number and seed size (Fig. 5D), negatively related to seed size (not shown). In forbs, the shoot density was positively related to the ratio between seed size and number of viable seeds per shoot (Fig. 5F).

## Discussion

### Response of grass and forb reproductive traits to fertilisation

The results reported here are novel, as for the first time the fertilisation effects on the reproduction behaviour were studied considering all main traits of the gamic reproduction and species of the grassland vegetation.

The overall size of the reproductive system (viable seed number per shoot) was significantly affected by fertilisation more frequently in grasses than in forbs (38% and 10% of species, respectively). However, in both functional groups, the traits more strongly and frequently affected were the number of inflorescences per shoot and the seed viability.

In grasses, this study highlighted a much greater fertilisation effect on the spikelet number per shoot than on the flower number per spikelet. Past studies in the field of seed propagation showed contrasting results, with cases of stronger responses for the former and the latter trait (e.g., in *Festuca pratensis* and *Lolium perenne*^27^). The relative fertilisation effect on the two traits is likely species-specific, but the greater effect on spikelet number per shoot found here may be dependent upon the intrinsic characteristics of permanent grasslands. Temperate perennial grasses are dual induction species, and the contribution to the seed production of the following year is highest for shoots that attained the maximum development (i.e., maximum number of buds at the shoot apex) during the late summer and autumn of the previous year^28^. The greater spikelet number per shoot observed here in the fertilized treatments was therefore likely to be due to the faster shoot development (i.e., more buds) induced by fertilisation during several months of the second part of the growing season, a period for grass shoot growth that is longer in permanent grasslands than in the fields cultivated for seed propagation.

In forbs, a positive fertilisation effect was found on both the inflorescence number per shoot (as in grasses) and the flower number per inflorescence. The positive effect on the number of inflorescences could be due to enhanced vegetative growth (i.e., high number of axillary buds) and increased floral differentiation (higher proportion of branches within an inflorescence) and was found in both legume and non-leguminous forbs^29,30^. Higher number of flowers per inflorescence were found in more fertile soils at both taxonomic and ecological levels. For example, the head size of different *Helianthus* species was shown to be directly related to the fertility of the typical species habitat^31^ (taxonomic level) and the flower number per inflorescence in plants from the same population was higher in *Ipomopsis aggregata*^32^, *Leucanthemum vulgare* and *Trifolium pratense*^33^ growing on more fertile soils (ecological level).

Seed quality traits were affected by fertilisation less frequently and to a lesser extent. than inflorescence size traits. OSU was, in most cases, independent of fertilisation. This result is not uncommon in seed propagation studies of grassland species (see results for *Lolium perenne*^8,34^), but is not consistent with the Stephenson’s^10^ general view of seed set being normally limited by resource availability. Instead, it is in agreement with the results of the Wiens’ study^11^, which found that resource limitations are critical for flower production, but seed set is genetically determined. For the species studied here, the flower number increased but OSU remained the same under the HighFert treatment.

Seed viability was more clearly affected by fertilisation than OSU. Discussing this result is difficult as to our knowledge, no studies have reported the effects of fertilisation on viability because many researchers consider this trait to be equivalent to or a proxy for germination, and viability is typically investigated through germinability test ^35^. However, as viability and germinability were found here to be strictly correlated (r^2^ = 0.83), it seems possible to compare the viability results obtained here with the germinability results from previous research. Past studies have reported positive, no, or negative fertilisation effects on germinability (see the review by Gray and Thomas^36^), as obtained here for viability. In the current study, the variation in fertilisation effects was found to be largely related to the breeding system, as in most cases viability increased in obligate or facultative autogamous species, particularly in the wind-pollinated grasses, whereas it decreased in allogamous species, particularly in insect-pollinated species. Past studies showed that fertilisation can improve seed germination by increasing the nutrient concentration in seeds (e.g., Cheplick and Sung^37^). This finding could explain the behaviour of most grasses, for which viability increased but does not explain the reduced viability of most forbs under HighFert. Two compatible hypotheses are possible to explain the result for forbs. The first refers directly to the allogamous character of the involved species. Studies have found that fertilisation can change pollen and nectar quality, reducing their attractiveness to pollinators^14^), or increasing the time spent by pollinators at each plant to collect the amino acid richer nectar^13^. Both fewer pollinator visits and longer time spent per plant resulted in increased self-fertilization with a consequent decrease in seed set and viability in allogamous species^13,14^. Higher selfing percentage resulted in lower seed viability in the anemophilous *Abies pinsapo*, too^38^. The second hypothesis posits that lower forb seed viability is due to competition from other species^39^. In our study, this competition could have come from grasses, which increased greatly in the fertilised treatments.

### Multispecies correlations among reproductive traits

All of the negative relationships found between the reproductive traits can be interpreted as trade-off behaviours due to allocation of resources to one function over another^40^. For both grasses and forbs, the primary differentiation in strategy was between species yielding many light seeds with low probability to produce seedlings able to win the competition of the established plants and species producing a few heavy seeds, whose seedlings had a higher probability of survival^41^ (Fig. 5D).

An effect of fertilisation on the described trade-offs is visible from the point distribution in the graphs 5A–5D but was never robust enough to reach the level of statistical significance. However, Fig. 5D details a result of particular interest: among the traits defining the primary trade-off strategy, i.e. seed number and seed size, the seed number underwent the highest positive changes due to fertilisation. This could imply a competitive advantage for grasses, which showed a greater increase in seed number per shoot following fertilisation than forbs.

No relationship was found between any reproductive trait and the shoot density in any species group at LowFert. This result confirms that species adapted to poor soils rely more on vegetative than sexual reproduction^42^. Instead, two significant, positive relationships were found only in HighFert between reproductive traits measured at different levels of fertilisation. The shoot density (population level) was positively related to the seed size in forbs (see also Scotton^20^) and to the number of viable seeds per shoot in grasses (shoot level). As seed production per shoot and seed size were strongly negatively correlated, the two relationships indicated that in HighFert the shoot density of the two functional groups was regulated in inverse ways by the seed number/seed size trade-off.

Forbs that thrived in HighFert had a high seed size, even if their seed production per shoot was lower. The main species behaving in this way were *Centaurea nigrescens*, *Knautia drymeia*, and *Rhinanthus freynii*. As previously indicated, larger seed size can increase species recruitment by increasing the probability of seedling survival in closed vegetation^41^. The result obtained here indicates that this is the case particularly in a fertilised meadow and for forbs, likely because forb seedlings from large seeds can better overcome the recruitment obstacle represented by the strong competitive ability of grass species in the high-N environments approximated by the HighFert treatment^43^. This finding does not negate the importance of seed production per shoot, because it increases the baseline number of seeds proceeding to the establishment stage. This would explain why for forbs the shoot density was found to be significantly related to the fertilisation-related increase of the number of viable seeds per shoot.

Contrary to forbs, grasses with the highest shoot density were characterised in HighFert by large numbers of viable seed per shoot. The main species displaying this behaviour were the light-seeded *Holcus lanatus*, *Poa trivialis*, and *Trisetum flavescens*. One possible explanation, supported by the results of both this study and that of Scotton^20^, is that a higher percentage of dormant seeds allows grasses to germinate and establish in autumn, the predominant time in which grass seeds germinate^44^. Strong subsequent tillering occurring under the mild autumn climate^5^ would allow many grass tillers to vernalise during winter and, therefore, enter the reproductive stage in the subsequent spring^28^. In autumn, when the growth of established vegetation is low, many grass seeds could successfully establish to the seedling stage, and the competitive advantage of large seeds would be less important. This effect could be reinforced by the soil surface covered by vegetation in the autumn (= space free for seedling establishment), being particularly low in fertilised meadows (e.g., D’Ottavio et al.^45^).

The relationships between reproductive traits and shoot density discussed above were not found for the low fertilisation level. This result suggests that on infertile soils with low above-ground biomass, competition from established plants is less of a problem and stress-tolerance against soil nutrient shortages becomes more important.

## Conclusions

Even if with considerable among-species variability, in most cases the size of inflorescences increased due to fertilisation in both species groups. Instead, the efficiency of the transformation of ovules to viable seeds generally increased in grasses but more often decreased in allogamous insect-pollinated forbs.

Fertilisation did not change the trade-off relationships between individual reproduction traits. However, our results showed that reproductive traits have presumably important relationship with the species composition of the grassland and that the effects of fertilisation on reproductive traits can be hypothesized to be involved in the often-observed vegetation change from a species-rich *Arrhenatherum elatius* meadow to a species-poor grassland with high grass abundance. In the treatments with no or little fertiliser, none of the considered traits was found to affect the shoot density in either functional group, confirming the relatively low importance of reproduction by seed in low soil fertility grasslands. At higher levels of soil fertility, significant but inverse relationships were found for the two species groups. The grasses with the higher shoot densities were those with the largest number of viable seeds produced per shoot and which increased more the production of viable seeds due to fertilisation. Instead, forbs with higher shoot density in the fertilised meadow were those with higher seed size. The higher dormancy of grass seeds and the lower soil vegetation cover of the fertilised meadows during the last part of the growing season likely explain the first result. The ability of forb seedlings from high-weight seeds to overcome the competition of grass was likely the mechanism behind the second result.

This study provides some information useful for nature conservation and ecological restoration. Species-rich grasslands are better maintained under no fertilisation because most insect-pollinated forbs have here higher seed viability and are not subject to a selection based on the seed size, as found in the presence of fertilisation. If both nature conservation and grassland utilisation for species-rich seed harvesting are the aims, an intermediate fertilisation treatment (011) becomes probably also important as it maintains most species of the no fertilisation treatment but also provides a higher seed production, allowing a more abundant harvest of seed to be used in the establishment of species-rich grasslands. This suggestion should, however, be further checked with an analysis to be performed at the whole meadow level.

## Supplementary Information

Supplementary Information.

## Data Availability

The raw data of the study can be found online within the supporting information of this paper (Annex 3).
