# Supplementary material for: Effects of fertilisation on grass and forb gamic reproduction in semi-natural grasslands
Source: Sci Rep. 2021 Sep 27;11:19146. doi: 10.1038/s41598-021-98756-5 (PMC8476543; doi:10.1038/s41598-021-98756-5)
Supplement: Supplementary file 1 — Supplementary Information. [file 41598_2021_98756_MOESM1_ESM.pdf]

Supplementary information

Annex 1. 2012-2017 mean species composition (columns 3-5) of the three fertilisation treatments surveyed for seed production at the end of the first growth in a grassland fertilization trial in the Italian eastern Pre-Alps. The values represent visual estimations of species percent abundance. Estimations smaller than 1 % are indicated with +. Species identification and names follow Pignatti (1982) (*). Reproductive biological traits of the studied species from BIOLFLOR (columns 4-6).

Species group (2)		SPECIES COMPOSITION			TRAITS OF REPRODUCTIVE BIOLOGY		
	Fertilisation treatment	000	011	222	Type of reproduction (3)	Breeding system (4)	Pollen vector (5)
	Grasses abundance (%)	30	39	76			
	Legume abundance (%)	8	32	3			
	Forb abundance (%)	62	29	21			
	Mean yearly no. of species on 24 m^2	30.3	29.6	20.5			
	Total no. of species surveyed in six years on 72 m^2	61	53	48			
3	Achillea roseoalba Ehrend.	+	1.0	+	SV	X	IN
1	Anthoxanthum odoratum L.	3.5	3.3	1.1	SV	X	WI
3	Centaurea nigrescens Willd.	1.5	1.4	+	S	X	IN
3	Cerastium fontanum subsp. vulgare (Hartm.) Greuter & Burdet	+	1.2	+	SV	A	IN
1	Cynosurus cristatus L.	+	1.9	+	S	X	WI
1	Festuca pratensis subsp. pratensis Huds.	+	+	+	S	X	WI
2	Trifolium pratense subsp. pratense L.	3.4	15.1	1.5	S	X	IN
1	Holcus lanatus L.	+	3.4	14.8	SV	X	WI
3	Salvia pratensis subsp. pratensis L.	4.8	+	+	S	X	IN
1	Trisetum flavescens subsp. flavescens (L.) P. Beauv.	+	9.7	15.5	S	X	WI
3	Clinopodium vulgare subsp. vulgare L.	+	+	+	SV	X	IN
3	Silene vulgaris subsp. vulgaris (Moench) Garcke	+	+	+	S	X	IN
3	Rhinanthus freynii (Sterneck) Fiori	+	+	+	S	AX	IN
3	Galium album subsp. album Mill.	1.0	2.9	11.2	.	.	.
1	Festuca rubra subsp. rubra L.	5.0	2.2	2.3	.	.	.
3	Ranunculus acris subsp. acris L.	+	1.8	+	S	X	IN
3	Plantago lanceolata L.	+	+	+	.	.	.
2	Lotus corniculatus L.	2.6	1.6	+	.	.	.
3	Leucanthemum vulgare aggr.	6.8	9.7	+	SV	AX	IN
3	Daucus carota L.	+	+	+	.	.	.
3	Prunella vulgaris L.	+	+	+	.	.	.
1	Poa pratensis L.	+	+	+	.	.	.
3	Campanula glomerata L.	+	+	+	.	.	.
3	Arabis hirsuta (L.) Scop.	+	+	+	.	.	.
3	Ranunculus tuberosus Lapeyr.	+	.	+	.	.	.
1	Brachypodium pinnatum (L.) P. Beauv.	7.2	.	.	SV	AX	WI
1	Briza media L.	4.6	+	.	SV	X	WI
3	Plantago media L.	2.5	+	+	SV	X	WI
3	Thymus pulegioides subsp. pulegioides L.	+	.	.	.	.	.
3	Polygala vulgaris subsp. vulgaris L.	+	.	.	.	.	.
1	Carex caryophyllea Latourr.	+	.	.	.	.	.
3	Orobanche gracilis Sm.	+	.	.	.	.	.
3	Bellis perennis L.	+	.	.	.	.	.
1	Bromus erectus subsp. erectus Huds.	+	.	.	.	.	.
1	Carex montana L.	+	.	.	.	.	.
3	Euphrasia rostkoviana subsp. rostkoviana Hayne	+	.	.	.	.	.
3	Potentilla erecta L. Raeusch.	+	.	.	.	.	.
3	Scabiosa columbaria subsp. columbaria L.	+	.	.	.	.	.
3	Tragopogon pratensis subsp. orientalis (L.) Celak	+	.	.	.	.	.
1	Helictotrichon pubescens subsp. pubescens (Huds.) Pilg.	1.2	2.2	+	SV	AX	WI
3	Knautia drymeia subsp. drymeia Heuff.	15.7	4.7	+	S	X	IN
3	Leontodon hispidus L.	24.1	1.4	+	SV	X	IN
3	Primula veris subsp. veris L.	+	+	.	SV	X	IN
1	Festuca rupicola Heuff.	2.2	+	.	S	X	WI
3	Stachys officinalis subsp. officinalis (L.) Trevis.	+	+	.	SV	A	IN
2	Medicago lupulina L.	1.5	1.4	+	SV	A	IN
3	Crepis biennis L.	+	+	.	.	.	.
1	Koeleria pyramidata (Lam.) P. Beauv.	+	+	.	.	.	.
1	Luzula campestris (L.) DC. in Lam & DC.	+	+	.	.	.	.
3	Alchemilla xanthochlora aggr.	+	+	.	.	.	.
3	Silene nutans subsp. nutans L.	+	+	.	.	.	.
3	Ajuga reptans L.	+	+	.	.	.	.
1	Dactylis glomerata subsp. glomerata L.	+	3.3	17.6	S	X	WI
1	Arrhenatherum elatius (L.) J. Presl & C. Presl	+	7.5	10.2	S	X	WI
1	Lolium perenne L.	+	2.8	2.6	S	X	WI
3	Rumex acetosa L.	+	+	2.1	SV	X	WI
1	Poa trivialis subsp. trivialis L.	+	2.3	9.5	SV	AX	WI
1	Carex spicata Huds.	.	+	+	S	AX	WI
1	Bromus hordeaceus subsp. hordeaceus L.	.	+	1.8	S	A	WI
2	Trifolium repens subsp. repens L.	+	14.5	1.4	.	.	.
3	Pimpinella major (L.) Huds.	+	1.0	+	.	.	.
3	Taraxacum officinale Weber	+	+	1.6	.	.	.
3	Veronica chamaedrys subsp. chamaedrys L.	+	+	+	.	.	.
3	Myosotis sylvatica Hoffm.	.	+	1.7	.	.	.
3	Silene flos-cuculi (L.) Clairv.	.	+	+	.	.	.
2	Trifolium dubium Sibth.	.	+	.	.	.	.
3	Rumex obtusifolius subsp. obtusifolius L.	.	.	+	.	.	.
3	Veronica arvensis L.	.	.	+	.	.	.
2	Vicia cracca subsp. cracca L.	.	.	+	.	.	.
2	Lathyrus pratensis L.	.	.	+	.	.	.
3	Myosotis arvensis Hill	.	.	+	.	.	.

LEGEND. (1) Pignatti S., 1982. Flora d'Italia. Edagricole, Bologna. (2) Species group: 1 graminoids; 2 legume forbs, 3 other forbs. (3) Type of reproduction: S by seed or mostly by seed, SV by seed and vegetatively. (4) Breeding system: X obligate or mainly outcrossing, AX both outcrossing and autogamous, A obligate autogamous (5) Pollen vector: WI wind, IN insect.

Annex 2. Summary of the statistical analyses performed.

Question	Analyzed variables (Y)	Explanatory variables (X)	Type of analysis	Note
**FIRST AIM: STUDYING THE FERTILIZATION EFFECT ON THE REPRODUCTIVE BEHAVIOUR OF INDIVIDUAL SPECIES AND THE TWO SPECIES FUNCTIONAL GROUPS OF GRASSES AND FORBS.**				
Does fertilization influence the mean value of the reproductive traits in individual species?	Yearly values of the main nine reproductive traits in fourteen grasses and seventeeen forbs surveyed in six years.	Fertilization treatment (levels 000, 011 and 222: two or three levels, depending on the species), year, and block.	Mixed linear models under the repeated measure approach and Tukey's tests.	Analysis performed for each species separately.
Does the frequency of the different fertilization effects on the mean values of the reproductive traits differ between grasses and forbs?	Frequency of no / positive / negative response of nine main reproductive traits to fertilization found in the previous analysis in the species functional groups of grasses and forbs.	Species functional group (grasses and forbs).	Chi-square tests.	Analysis considering grasses and forbs together.
Does the mean values of the reproductive traits differ between grasses and forbs?	Multi-year mean of the main nine reproductive traits for each species.	Species functional group (grasses and forbs), fertilization level, and block (species considered as replicate within the species group, therefore not included as class factor).	General linear models and Tukey's tests.	Analysis considering grasses and forbs together.
Does the mean grass and forb value of the main nine reproductive traits differ between fertilization levels?	Multi-year mean of the main nine reproductive traits for each species.	Fertilization level, species, and block.	General linear models and Tukey's tests.	Analysis performed for grasses and forbs separately.
**SECOND AIM: FINDING SPECIES BIOLOGICAL AND ECOLOGICAL TRAITS EXPLAINING THEIR RESPONSE TO FERTILIZATION.**				
Is the variation of the reproductive traits due to fertilization related to the mean values of the other traits or to the ecological species behaviour?	Percent variation of two reproductive traits (number of ovules and viable seeds per shoot) in Highfert compared to Lowfert calculated for each species.	For each species, the average values of the nine main reproductive traits, the seven Ellenberg biondicator, and the percent variation of fertile shoot density.	Simple linear regressions.	Analysis performed for grasses and forbs separately or together.
Is the percent variation of the reproductive traits due to fertilization related to the species functional group or the species reproductive biology?	Percent variation of four reproductive traits (OSU, seed germinability, viability and weight) in Highfert compared to Lowfert calculated for each species.	Species group and three traits of the species reproduction biology (reproduction type, breeding system and pollen vector).	Parametric analysis of variance.	Analysis performed separately for each explanatory variable and considering grasses and forbs together.
**THIRD AIM: IDENTIFYING MULTISPECIES CORRELATIONS AMONG REPRODUCTIVE TRAITS AND THE POSSIBLE EFFECTS OF FERTILIZATION ON THEIR PATTERNS.**				
Are the species values of one reproductive trait related to the other reproductive traits or the fertile shoot density of the same species?	Multi-year mean of the main nine reproductive traits calculated for each species.	The multi-year meand of the other eight main reproductive traits and the fertile shoot density.	Standardized major axis regressions.	Analysis performed for grasses and forbs separately or together and for fertilisation levels separately or averaged.

Annex 3. Mean values of the reproductive traits of thirty-two grassland species studied in a fertilisation trial in the eastern Italian Alps. The effects of fertilisation and year are also shown.

Species functio-nal group	Species (1)	Growth period	Mean fertilis. Treatment (2)	Difference among means (3)	No. simple inflorescences per shoot		No. flowers per simple inflorescence		No. ovules per ovary		No. ovules per shoot		No. viable seeds per shoot		OSU		1000 seeds weigth		Seed viability %		Seed germinability %	
Grasses	AnOd	1	000	.	23.67	c	1	-	1	-	23.7	c	15.2	c	0.785	b	0.644	a	88.5	a	64.0	a
		1	011	.	32.97	b	1	-	1	-	33.0	b	23.9	b	0.896	a	0.639	a	88.2	a	68.7	a
		1	222	.	46.62	a	1	-	1	-	46.6	a	34.6	a	0.915	a	0.622	a	85.8	a	67.6	a
		1	P level (4)	>, =, < (5)	<0.0001	>	-	-	-	-	<0.0001	>	<0.0001	>	0.001	>	ns	=	ns	=	ns	=
	ArEl	1	000	.	-	-	-	-	-	-	-	-	-	-	-	-	-	-	-	-	-	-
		1	011	.	34.01	a	1	-	1	-	34.0	a	15.3	a	0.932	a	3.174	a	43.8	a	18.1	a
		1	222	.	38.01	a	1	-	1	-	38.0	a	12.3	a	0.839	b	2.802	b	42.3	a	17.7	a
		1	P level (4)	>, =, < (5)	ns	=	-	-	-	-	ns	=	ns	=	0.006	<	0.004	<	ns	=	ns	=
	AvPu	1	000	.	16.19	a	3.01	a	1	-	49.4	a	3.4	b	0.314	a	2.346	a	38.7	b	8.5	b
		1	011	.	16.64	a	3.05	a	1	-	50.7	a	6.2	a	0.367	a	2.316	a	51.8	a	20.4	a
		1	222	.	-	-	-	-	-	-	-	-	-	-	-	-	-	-	-	-	-	-
		1	P level (4)	>, =, < (5)	ns	=	ns	=	-	-	ns	=	0.0	>	ns	=	ns	=	0.022	>	0.004	>
	BrPi	1	000	.	8.08	a	8.01	a	1	-	64.2	a	2.8	a	0.527	a	1.260	b	20.6	b	8.0	b
		1	011	.	8.33	a	6.12	b	1	-	51.0	a	5.9	a	0.491	a	2.998	a	47.2	a	28.3	a
		1	222	.	-	-	-	-	-	-	-	-	-	-	-	-	-	-	-	-	-	-
		1	P level (4)	>, =, < (5)	ns	=	0.01	<	-	-	ns	=	ns	=	ns	=	0.002	>	0.002	>	0.000	>
	BrMe	1	000	.	26.04	-	3.78	-	1	-	99.1	-	20.8	-	0.441	-	0.453	-	66.9	-	19.0	-
		1	011	.	-	-	-	-	-	-	-	-	-	-	-	-	-	-	-	-	-	-
		1	222	.	-	-	-	-	-	-	-	-	-	-	-	-	-	-	-	-	-	-
		1	P level (4)	>, =, < (5)	-	-	-	-	-	-	-	-	-	-	-	-	-	-	-	-	-	-
	BrHo	1	000	.	-	-	-	-	-	-	-	-	-	-	-	-	-	-	-	-	-	-
		1	011	.	9.60	b	4.14	b	1	-	41.2	b	26.7	b	0.865	a	4.019	a	84.5	b	73.0	a
		1	222	.	12.84	a	4.86	a	1	-	62.0	a	40.0	a	0.801	b	3.970	a	92.8	a	78.3	a
		1	P level (4)	>, =, < (5)	<0.0001	>	0.01	>	-	-	0.0	>	<0.0001	>	0.014	<	ns	=	0.007	>	ns	=
	CaCo	1	000	.	-	-	-	-	-	-	-	-	-	-	-	-	-	-	-	-	-	-
		1	011	.	6.06	a	3.80	a	1	-	22.7	a	9.5	a	0.915	a	2.068	a	52.4	a	19.2	a
		1	222	.	6.10	a	4.07	a	1	-	24.4	a	11.5	a	0.847	b	1.870	b	59.2	a	22.7	a
		1	P level (4)	>, =, < (5)	ns	=	ns	=	-	-	ns	=	ns	=	0.006	<	<0.0001	<	ns	=	ns	=
	CyCr	1	000	.	42.12	b	2.79	a	1	-	117.2	b	55.0	b	0.657	b	0.680	a	91.6	a	76.0	a
		1	011	.	44.92	b	2.77	a	1	-	125.9	b	77.0	a	0.757	a	0.675	a	93.0	a	65.4	a
		1	222	.	57.17	a	2.87	a	1	-	165.0	a	97.1	a	0.774	a	0.706	a	87.1	a	48.0	b
		1	P level (4)	>, =, < (5)	<0.0001	>	ns	=	-	-	<0.0001	>	<0.0001	>	0.006	>	ns	=	ns	=	0.002	<
	DaGl	1	000	.	59.95	c	3.67	a	1	-	226.2	c	69.9	a	0.689	b	0.856	b	50.4	a	11.1	b
		1	011	.	75.57	b	3.62	a	1	-	275.0	b	98.8	a	0.772	a	0.868	ab	47.7	a	23.9	a
		1	222	.	97.63	a	3.75	a	1	-	372.8	a	114.2	a	0.682	b	0.957	a	54.5	a	33.5	a
		1	P level (4)	>, =, < (5)	<0.0001	>	ns	=	-	-	<0.0001	>	ns	=	0.007	=	0.042	>	ns	=	0.015	>
	FePr	1	000	.	14.48	a	4.99	a	1	-	69.1	b	13.4	a	0.448	a	2.150	a	74.7	a	52.7	a
		1	011	.	13.77	a	5.19	a	1	-	72.3	b	16.2	a	0.478	a	2.260	a	72.7	ab	53.4	a
		1	222	.	15.75	a	5.51	a	1	-	87.9	a	19.4	a	0.492	a	2.029	a	63.2	b	37.1	b
		1	P level (4)	>, =, < (5)	0.03	=	ns	=	-	-	0.0	>	ns	=	ns	=	ns	=	0.037	<	0.017	<
	FeRu	1	000	.	11.74	b	4.62	a	1	-	53.5	b	6.0	a	0.420	a	0.696	a	51.8	a	20.3	a
		1	011	.	14.01	a	4.71	a	1	-	66.3	a	7.3	a	0.421	a	0.716	a	50.3	a	18.3	a
		1	222	.	-	-	-	-	-	-	-	-	-	-	-	-	-	-	-	-	-	-
		1	P level (4)	>, =, < (5)	0.02	>	ns	=	-	-	0.0	>	ns	=	ns	=	ns	=	ns	=	ns	=
	HoLa	1	000	.	94.24	b	1	-	1	-	89.8	b	46.4	b	0.818	a	0.264	a	76.0	a	46.7	a
		1	011	.	159.32	a	1	-	1	-	159.3	a	70.4	a	0.717	a	0.305	a	77.3	a	53.3	a
		1	222	.	180.03	a	1	-	1	-	180.0	a	79.8	a	0.692	b	0.308	a	80.3	a	58.7	a
		1	P level (4)	>, =, < (5)	<0.0001	>	-	-	-	-	<0.0001	>	0.0	>	0.040	<	ns	=	ns	=	ns	=
	LoPe	1	000	.	-	-	-	-	-	-	-	-	-	-	-	-	-	-	-	-	-	-
		1	011	.	11.54	b	4.18	b	1	-	48.0	b	7.2	b	0.356	b	1.929	a	73.9	a	46.3	a
		1	222	.	12.49	a	5.40	a	1	-	67.4	a	16.1	a	0.471	a	1.928	a	82.1	a	59.6	a
		1	P level (4)	>, =, < (5)	0.02	>	<0.0001	>	-	-	<0.0001	>	<0.0001	>	<0.0001	>	ns	=	ns	=	ns	=
	PoTr	1	000	.	-	-	-	-	-	-	-	-	-	-	-	-	-	-	-	-	-	-
		1	011	.	97.49	b	2.13	a	1	-	197.0	b	48.3	b	0.711	a	0.210	b	39.3	a	11.3	a
		1	222	.	173.00	a	2.12	a	1	-	359.9	a	82.7	a	0.635	a	0.226	a	48.6	a	4.9	a
		1	P level (4)	>, =, < (5)	<0.0001	>	ns	=	-	-	<0.0001	>	0.0	>	ns	=	0.016	>	ns	=	ns	=
	TrFl	1	000	.	119.07	b	2.37	a	1	-	280.0	b	70.0	a	0.567	a	0.245	a	60.1	a	31.1	a
		1	011	.	120.12	b	2.46	a	1	-	295.5	b	84.9	a	0.659	a	0.217	b	53.7	a	21.1	a
		1	222	.	159.57	a	2.31	a	1	-	362.6	a	124.0	a	0.629	a	0.224	b	59.1	a	29.7	a
		1	P level (4)	>, =, < (5)	0.00	>	ns	=	-	-	0.0	>	ns	=	ns	=	0.003	<	ns	=	ns	=
Forbs	AcRo	1	000	.	17.63	b	15.94	a	1	-	281.6	b	112.6	a	0.603	a	0.127	b	64.2	a	41.9	a
		1	011	.	24.18	b	15.94	a	1	-	372.8	b	122.0	a	0.628	a	0.126	b	59.2	a	43.8	a
		1	222	.	32.03	a	16.12	a	1	-	490.1	a	95.4	a	0.587	a	0.137	a	47.1	b	39.2	a
		1	P level (4)	>, =, < (5)	0.00	>	ns	=	-	-	0.0	>	ns	=	ns	=	0.002	>	0.001	<	ns	=
		2	000	.	10.65	b	14.56	a	1	-	148.6	b	47.9	a	0.651	b	0.135	b	56.3	a	33.1	a
		2	011	.	18.89	a	16.70	a	1	-	319.2	a	93.3	a	0.811	a	0.150	a	46.3	a	30.4	a
		2	222	.	21.03	a	15.72	a	1	-	308.7	a	101.7	a	0.772	a	0.128	b	38.2	a	21.4	a
		2	P level (4)	>, =, < (5)	0.00	>	ns	=	-	-	0.0	>	ns	=	0.002	>	<0.0001	=	ns	=	ns	=
	RhFr	1	000	.	4.03	b	5.73	a	6.58465	a	143.7	a	16.8	a	0.843	a	1.887	a	14.6	a	0	a
		1	011	.	4.57	ab	5.20	a	6.62038	a	151.3	a	17.0	a	0.932	a	1.916	a	17.5	a	0.1	a
		1	222	.	5.95	a	6.10	a	5.95722	a	190.5	a	4.5	a	0.898	a	1.932	a	8.2	a	0	a
		1	P level (4)	>, =, < (5)	0.02	>	ns	=	ns	=	ns	=	ns	=	ns	=	ns	=	ns	=	ns	=
	RuAc	1	000	.	-	-	-	-	-	-	-	-	-	-	-	-	-	-	-	-	-	-
		1	011	.	9.40	a	47.95	a	1	-	464.4	a	287.1	a	0.814	a	0.692	a	88.3	a	83.1	a
		1	222	.	8.37	a	44.41	a	1	-	393.1	a	257.5	a	0.820	a	0.782	a	84.8	a	74.9	a
		1	P level (4)	>, =, < (5)	ns	=	ns	=	-	-	ns	=	ns	=	ns	=	ns	=	ns	=	ns	=
	SaPr	1	000	.	1.60	a	38.09	a	4	-	241.8	a	58.8	a	0.639	a	1.359	a	43.0	a	34.7	a
		1	011	.	2.01	a	43.94	a	4	-	362.5	a	59.6	a	0.614	a	1.225	a	36.8	a	30.8	a
		1	222	.	2.10	a	35.89	a	4	-	288.6	a	38.3	a	0.572	a	1.326	a	32.5	a	25.2	a
		1	P level (4)	>, =, < (5)	ns	=	ns	=	-	-	ns	=	ns	=	ns	=	ns	=	ns	=	ns	=
	CeNi	1	000	.	1.93	b	47.28	b	1	-	91.3	c	18.8	a	0.466	a	1.702	b	50.6	a	46.6	a
		1	011	.	3.32	a	47.66	b	1	-	154.4	b	16.7	a	0.318	b	1.865	a	48.8	a	35.0	b
		1	222	.	3.70	a	57.96	a	1	-	219.9	a	20.4	a	0.329	b	1.670	b	44.5	a	33.0	b
		1	P level (4)	>, =, < (5)	<0.0001	>	0.00	>	-	-	<0.0001	>	ns	=	<0.0001	<	0.001	=	ns	=	0.0	<
		2	000	.	3.00	b	45.65	b	1	-	138.3	b	17.9	a	0.379	a	1.644	a	65.1	a	51.4	a
		2	011	.	3.47	b	56.58	a	1	-	198.6	ab	12.3	a	0.316	a	1.660	a	40.8	b	28.5	b
		2	222	.	4.35	a	51.09	ab	1	-	226.8	a	19.2	a	0.356	a	1.739	a	38.7	b	24.6	b
		2	P level (4)	>, =, < (5)	0.01	>	0.01	=	-	-	0.0	>	ns	=	ns	=	ns	=	<0.0001	<	0.0	<
	ClVu	1	000	.	1.27	b	20.84	b	4	-	102.9	b	45.6	a	0.624	a	0.440	a	90.6	a	79.7	a
		1	011	.	1.53	ab	20.56	b	4	-	123.9	b	44.5	a	0.710	a	0.392	ab	67.3	a	42.9	b
		1	222	.	2.01	a	31.99	a	4	-	255.6	a	103.8	a	0.685	a	0.362	b	66.6	a	59.1	b
		1	P level (4)	>, =, < (5)	0.00	>	0.00	>	-	-	0.0	>	ns	=	ns	=	0.016	<	ns	=	0.0	<
		2	000	.	1.62	a	14.81	b	4	-	99.7	a	30.1	a	0.664	a	0.318	a	57.1	a	43.4	a
		2	011	.	2.06	a	15.75	b	4	-	130.1	a	38.7	a	0.565	a	0.345	a	65.1	a	59.9	a
		2	222	.	2.01	a	19.33	a	4	-	151.0	a	25.4	a	0.640	a	0.272	b	28.9	b	20.8	b
		2	P level (4)	>, =, < (5)	ns	=	0.00	>	-	-	ns	=	ns	=	ns	=	0.001	<	0.0011	<	0.0004	<
	KnDr	1	000	.	2.50	a	21.25	a	1	-	50.5	a	9.9	a	0.565	a	2.212	b	35.6	a	0.1	b
		1	011	.	2.74	a	16.52	b	1	-	45.2	a	9.6	a	0.577	a	2.644	a	28.0	a	0.6	a
		1	222	.	-	-	-	-	-	-	-	-	-	-	-	-	-	-	-	-	-	-
		1	P level (4)	>, =, < (5)	ns	=	0.00	<	-	-	ns	=	ns	=	ns	=	<0.0001	>	ns	=	0.0252	>
	MeLu	1	000	.	9.39	b	9.38	a	1	-	85.3	b	30.7	a	1.000	a	1.474	b	33.8	a	25.2	a
		1	011	.	14.70	b	11.44	a	1	-	162.6	b	104.1	a	0.931	b	1.832	a	67.4	a	64.7	a
		1	222	.	23.42	a	13.85	a	1	-	207.0	a	78.1	a	0.977	ab	1.854	a	80.6	a	74.3	a
		1	P level (4)	>, =, < (5)	0.00	>	ns	=	-	-	0.0	>	ns	=	0.003	=	0.001	>	ns	=	ns	=
	StOf	1	000	.	4.25	b	11.13	a	4	-	191.6	a	10.4	a	0.573	a	0.674	a	15.1	a	0.8	b
		1	011	.	4.99	a	9.82	b	4	-	194.8	a	24.1	a	0.597	a	0.505	b	22.5	a	13.1	a
		1	222	.	-	-	-	-	-	-	-	-	-	-	-	-	-	-	-	-	-	-
		1	P level (4)	>, =, < (5)	0.02	>	0.05	<	-	-	ns	=	ns	=	ns	=	0.0003	<	ns	=	0.0209	>
	TrPr	1	000	.	1.47	a	33.31	b	2	-	98.9	b	9.4	a	0.209	a	1.237	a	92.7	a	78.8	a
		1	011	.	1.50	a	44.74	a	2	-	130.1	a	10.8	a	0.201	a	1.350	a	87.0	a	70.6	a
		1	222	.	1.66	a	39.85	a	2	-	125.9	a	11.0	a	0.199	a	1.285	a	82.6	a	72.4	a
		1	P level (4)	>, =, < (5)	ns	=	0.00	>	-	-	0.0	>	ns	=	ns	=	ns	=	ns	=	ns	=
		2	000	.	1.62	ab	44.03	a	2	-	143.9	ab	15.6	a	0.244	a	1.071	a	85.3	a	68.6	a
		2	011	.	1.57	b	39.89	a	2	-	123.0	b	15.0	a	0.240	a	0.948	a	83.5	a	60.1	a
		2	222	.	1.94	a	43.18	a	2	-	167.5	a	15.9	a	0.235	a	0.969	a	83.5	a	72.2	a
		2	P level (4)	>, =, < (5)	0.03	>	ns	=	-	-	0.0	=	ns	=	ns	=	ns	=	ns	=	ns	=
	LeHi	2	000	.	1	-	64.36	b	1	-	64.4	b	28.8	a	0.639	a	0.826	a	79.3	a	58.6	b
		2	011	.	1	-	80.79	a	1	-	80.8	a	28.7	a	0.568	a	0.885	a	77.7	a	69.1	a
		2	222	.	-	-	-	-	-	-	-	-	-	-	-	-	-	-	-	-	-	-
		2	P level (4)	>, =, < (5)	-	-	0.00	>	-	-	0.0	>	ns	=	ns	=	ns	=	ns	=	0.014	>
	LeVu	1	000	.	1	-	120.20	b	1	-	120.2	b	85.9	b	0.997	a	0.413	a	73.0	a	63.0	a
		1	011	.	1	-	204.51	a	1	-	204.5	a	175.5	a	0.910	a	0.334	a	96.3	a	95.0	a
		1	222	.	-	-	-	-	-	-	-	-	-	-	-	-	-	-	-	-	-	-
		1	P level (4)	>, =, < (5)	-	-	0.02	>	-	-	0.0	>	0.0	>	ns	=	ns	=	ns	=	ns	=
	PlMe	2	000	.	1	-	104.32	a	2.46531	a	254.5	a	125.8	a	0.834	a	0.416	a	56.3	a	27.4	a
		2	011	.	1	-	122.44	a	2.75063	a	326.2	a	95.9	a	0.655	a	0.396	a	45.8	a	18.6	a
		2	222	.	-	-	-	-	-	-	-	-	-	-	-	-	-	-	-	-	-	-
		2	P level (4)	>, =, < (5)	-	-	ns	=	ns	=	ns	=	ns	=	ns	=	ns	=	ns	=	ns	=
	PrVe	1	000	.	1	-	6.38	a	36.6282	a	232.2	a	136.1	a	0.813	a	0.668	a	86.1	a	0	a
		1	011	.	1	-	7.42	a	35.7068	a	266.6	a	144.3	a	0.735	a	0.670	a	88.8	a	1.5	a
		1	222	.	-	-	-	-	-	-	-	-	-	-	-	-	-	-	-	-	-	-
		1	P level (4)	>, =, < (5)	-	-	ns	=	ns	=	ns	=	ns	=	ns	=	ns	=	ns	=	ns	=
	CeFo	1	000	.	-	-	-	-	22.1733	a	123.2	b	24.3	b	0.468	b	0.156	a	66.4	a	51.4	a
		1	011	.	-	-	-	-	22.9804	a	172.2	a	32.0	ab	0.402	b	0.155	a	70.4	a	53.3	a
		1	222	.	-	-	-	-	23.6456	a	183.8	a	50.2	a	0.553	a	0.138	a	67.1	a	58.0	a
		1	P level (4)	>, =, < (5)	-	-	-	-	ns	=	0.0	>	0.0	>	0.031	>	ns	=	ns	=	ns	=
	RaAc	2	000	.	-	-	-	-	23.0494	a	112.2	a	32.7	a	0.731	b	1.161	a	32.1	a	2.4	a
		2	011	.	-	-	-	-	23.1582	a	128.1	a	19.5	a	0.757	b	0.690	c	23.3	a	0	a
		2	222	.	-	-	-	-	17.037	b	112.0	a	13.6	a	0.887	a	0.889	b	16.9	a	4.6	a
		2	P level (4)	>, =, < (5)	-	-	-	-	0.0001	<	ns	=	ns	=	0.014	>	<0.0001	<	ns	=	ns	=
	SiVu	1	000	.	-	-	-	-	53.1018	b	211.7	b	20.7	b	0.294	a	0.491	b	71.4	b	67.2	b
		1	011	.	-	-	-	-	77.1142	a	430.8	a	40.5	b	0.215	a	0.578	ab	88.5	a	88.0	a
		1	222	.	-	-	-	-	62.3295	b	401.4	a	71.6	a	0.348	a	0.632	a	95.5	a	94.2	a
		1	P level (4)	>, =, < (5)	-	-	-	-	<0.0001	=	<0.0001	>	<0.0001	>	ns	=	0.009	>	0.0045	>	0.0011	>

Legend.

(1) Grasses:AnOd, Anthoxanthum odoratum; ArEl, Arrhenatherum elatius; AvPu, Avenula pubescens; BrPi, Brachypodium rupestre; BrMe, Briza media; BrHo, Bromus hordeaceus; CaCo, Carex contigua; CyCr, Cynosurus cristatus; DaGl, Dactylis glomerata; FePr, Festuca pratensis; FeRu, Festuca rupicola; HoLa, Holcus lanatus; LoPe, Lolium perenne; PoTr, Poa trivialis; TrFl, Trisetum flavescens; Forbs; AcRo, Achillea roseo-alba; CeNi, Centaurea nigrescens; CeFo, Cerastium fontanum; ClVu, Clinopodium vulgare; KnDr, Knautia drymeia; LeHi, Leontodon hispidus; LeVu, Leucanthemum vulgare; MeLu, Medicago lupulina; PlMe, Plantago media; PrVe, Primula veris; RaAc, Ranunculus acris; RhFr, Rhinanthus freynii; RuAc, Rumex acetosa; SaPr, Salvia pratensis; SiVu, Silene vulgaris; StOf, Stachys officinalis; TrPr, Trifolium pratense.

(2) Fertilisation treatment: see main text. (3) Difference among means: means with no letter in common are different at least at p≤0.05. (4) P level of the Linear mixed model. (5) >, =, <: trait mean in HighFert higher, equal or lower than in LowFert.
